# Seroprevalence of Severe Acute Respiratory Syndrome Coronavirus 2 IgG in Juba, South Sudan, 2020^1^

**DOI:** 10.3201/eid2706.210568

**Published:** 2021-06

**Authors:** Kirsten E. Wiens, Pinyi Nyimol Mawien, John Rumunu, Damien Slater, Forrest K. Jones, Serina Moheed, Andrea Caflisch, Bior K. Bior, Iboyi Amanya Jacob, Richard Lino Lako, Argata Guracha Guyo, Olushayo Oluseun Olu, Sylvester Maleghemi, Andrew Baguma, Juma John Hassen, Sheila K. Baya, Lul Deng, Justin Lessler, Maya N. Demby, Vanessa Sanchez, Rachel Mills, Clare Fraser, Richelle C. Charles, Jason B. Harris, Andrew S. Azman, Joseph F. Wamala

**Affiliations:** Johns Hopkins Bloomberg School of Public Health, Baltimore, Maryland, USA (K.E. Wiens, F.K. Jones, J. Lessler, M.N. Demby, A.S. Azman);; Republic of South Sudan Ministry of Health, Juba, South Sudan (P.N. Mawien, J. Rumunu, B.K. Bior, I.A. Jacob, R.L. Lako, L. Deng);; Massachusetts General Hospital, Boston, Massachusetts, USA (D. Slater, S. Moheed, V. Sanchez, R. Mills, C. Fraser, R.C. Charles, J.B. Harris);; International Organization for Migration, Juba (A. Caflisch);; World Health Organization, Juba (A.G. Guyo, O.O. Olu, S. Maleghemi, A. Baguma, J.J. Hassen, S.K. Baya, J.F. Wamala);; Kabale University School of Medicine, Kabale, Uganda (A. Baguma);; Harvard Medical School, Boston (R.C. Charles, J.B. Harris);; Médecins Sans Frontières, Geneva, Switzerland (A.S. Azman);; Institute of Global Health, Geneva (A.S. Azman)

**Keywords:** antibodies, coronavirus disease, COVID-19, influenza, Juba, respiratory infections, SARS-CoV-2, seroprevalence, serosurvey, South Sudan, sub-Saharan Africa, severe acute respiratory syndrome coronavirus 2, viruses

## Abstract

Relatively few coronavirus disease cases and deaths have been reported from sub-Saharan Africa, although the extent of its spread remains unclear. During August 10–September 11, 2020, we recruited 2,214 participants for a representative household-based cross-sectional serosurvey in Juba, South Sudan. We found 22.3% of participants had severe acute respiratory syndrome coronavirus 2 (SARS-CoV-2) receptor binding domain IgG titers above prepandemic levels. After accounting for waning antibody levels, age, and sex, we estimated that 38.3% (95% credible interval 31.8%–46.5%) of the population had been infected with SARS-CoV-2. At this rate, for each PCR–confirmed SARS-CoV-2 infection reported by the Ministry of Health, 103 (95% credible interval 86–126) infections would have been unreported, meaning SARS-CoV-2 has likely spread extensively within Juba. We also found differences in background reactivity in Juba compared with Boston, Massachusetts, USA, where the immunoassay was validated. Our findings underscore the need to validate serologic tests in sub-Saharan Africa populations.

Globally, >100 million cases and >2.6 million deaths had been attributed to coronavirus disease (COVID-19) as of March 14, 2021 (*1*). Most cases have been reported in Europe and the Americas. In Africa, >2.9 million cases and ≈75,000 deaths have been reported (*1*). Reasons for the lower reported incidence and death associated with COVID-19 in Africa during the first 6–8 months of the pandemic are unclear but may include differences in age distribution, immune history, climate, early mitigation measures, and epidemiologic connectivity between geographic regions (*2*,*3*). However, our understanding of the true spread of severe acute respiratory virus coronavirus 2 (SARS-CoV-2) has been obscured by limited testing capabilities, underreported deaths, and undetected mild or asymptomatic infections (*4*). Population-based serological surveys, hundreds of which have been conducted worldwide, can help shed light on the extent of this underestimation of SARS-CoV-2 infections (*5*,*6*). As of March 18, 2021, only 16 studies published or available in preprint had been conducted in sub-Saharan Africa (*7**–**16*; H. Majiya et al., unpub. data, https://doi.org/10.1101/2020.08.04.20168112; B.N. Alemu et al., unpub. data, https://doi.org/10.1101/2020.10.13.337287; O. Ige et al., unpub. data, https://doi.org/10.1101/2020.11.24.20231324; I.M.O. Adetifa et al., unpub. data, https://doi.org/10.1101/2021.02.09.21251404; R. Lucinde et al., unpub. data, https://doi.org/10.1101/2021.02.05.21250735; E.W. Kagucia et al., unpub. data, https://doi.org/10.1101/2021.02.12.21251294; M.J. Peluso et al., unpub. data, https://doi.org/10.1101/2021.03.03.21251639). Only 3 of those reports (from Nigeria, Ethiopia, and Zambia) were population-based representative studies. No serosurveys had been conducted in South Sudan. 

South Sudan confirmed its first COVID-19 case in the capital, Juba, on April 4, 2020 (*17*), and saw its first wave of reported cases during May–July 2020 (Figure 1). By August 31, 2020, a total of 1,873 virologically confirmed SARS-CoV-2 infections (≈47/10,000 residents) had been reported from 18,156 reverse transcription PCR (RT-PCR) tests conducted in Juba. RT-PCR testing in South Sudan, including Juba, has remained limited because of scarce reagents, few testing sites, limited willingness to be tested, and logistic challenges. Thus, as in much of sub-Saharan Africa, the true extent of SARS-CoV-2 spread in the population remains unknown. 

**Figure 1:**
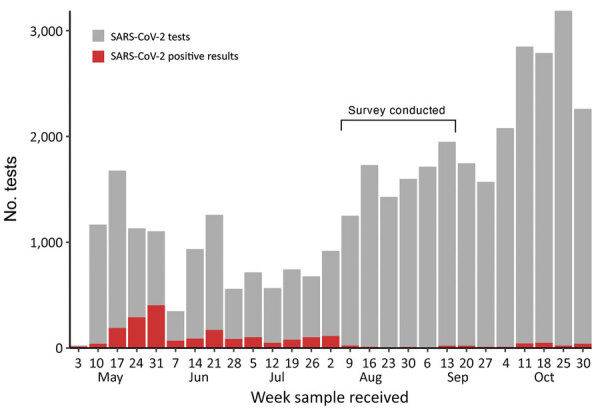
Number of weekly SARS-CoV-2 tests and infections reported in Juba, South Sudan, May 3–October 30, 2020. The survey of seroprevalence of SARS-CoV-2 IgG was conducted August 10–September 11. First coronavirus disease case in South Sudan was identified on April 2 and confirmed on April 4, 2020 (*23*). SARS-CoV-2, severe acute respiratory syndrome coronavirus 2.

Understanding SARS-CoV-2 spread is particularly important for guiding COVID-19 mitigation efforts in light of South Sudan’s complex humanitarian and public health context. South Sudan has experienced years of conflict, leading to 1.61 million internally displaced persons (IDP). Severe food insecurity affects more than half the population: 6 million people, including 1.3 million malnourished children (*18*,*19*). In Juba, 28.7% of households indicated that they were unable to access health care services when needed in the first 6 months of the pandemic; this number increased to 43.2% among residents in the lowest wealth quintile (*20*). These underlying vulnerabilities may increase risk of SARS-CoV-2 spread and may themselves be compounded by direct and indirect effects of the epidemic.

To estimate the seroprevalence of SARS-CoV-2 antibodies and associated risk factors in Juba, we conducted a representative household-based cross-sectional serosurvey. Here we present the results of this serosurvey and discuss the implications for SARS-CoV-2 surveillance in South Sudan, as well as more broadly for serologic studies conducted in Africa and worldwide.

## Methods

### Study Design and Participants

We conducted a cross-sectional serosurvey in residential neighborhoods of the city of Juba and Juba County according to protocols from the World Health Organization’s Unity Studies (*5*). We determined urban demarcation based on residentially developed areas, local administrative boundaries, and existing transportation networks within the Northern Bari, Munuki, Juba, Kator, Rejaf, and Gondokoro payams (subcounty administrative divisions). Residents of Juba IDP camps I and III, former United Nations Mission in the Republic of South Sudan (UNMISS) civilian protection sites, were not included in the sampling frame.

The survey employed 2-stage cluster sampling. We used enumeration areas (EAs) as clusters and selected them using probability proportional to size sampling. We calculated probabilities based on the number of structures in the EA found by satellite imagery; we removed nonresidential areas that were mapped by field teams during a preliminary assessment. Within each sampled EA, we randomly selected 11 residential structures as households to recruit into the study. The target sample size was 2,750 (50 clusters of 55 respondents each), but 11.1% of the original 550 households declined to participate. The main reasons reported were stigma, fear of testing positive, fear that the health worker taking the sample would infect the participant, and concern about samples being taken abroad for analysis. Alternate households were randomly sampled using the same procedure as for the original households. Three initially selected EAs, inhabited by families of military personnel, were inaccessible and therefore we replaced them by randomly sampling new EAs from the same stratum.

We defined a household as a group of persons who slept under the same roof most nights and shared a cooking pot. Regardless of current or past COVID-19 illness, all household members were eligible for inclusion if they or their guardian provided written consent to participate and they were >1 year of age and had lived in the area >1 week before the survey. For households with >10 persons, only first-degree relatives of the head of household were eligible for study inclusion. If multiple households lived in 1 shelter, we blindly drew from labeled papers to randomly select 1 household for inclusion.

We interviewed eligible participants to collect information about sociodemographic characteristics, history of respiratory symptoms, SARS-CoV-2 tests, exposure risks in the previous 2 weeks, and all household deaths. We collected dried blood samples by drawing blood by lancet from a finger, heel, or toe, and applying a few drops onto Whatman 903 (https://www.cytivalifesciences.com) or Ahlstrom grade 226 filter paper (https://www.ahlstrom-munksjo.com). The blood was allowed to thoroughly saturate the paper and air dry overnight at ambient temperature. We stored these dried blood spot (DBS) samples in low gas-permeable plastic bags with desiccant added to reduce humidity and transported the samples at ambient temperature to Massachusetts General Hospital (Boston, MA, USA) according to IATA protocol, where they were stored at 4°C until tested. The South Sudan Ministry of Health Ethics Review Board approved the study protocol.

### Laboratory Analysis

DBS were eluted and tested for the presence of SARS-CoV-2 IgG targeting the receptor-binding domain (RBD) of the spike protein of SARS-CoV-2 using a quantitative ELISA previously developed and validated at Massachusetts General Hospital (*21*). The assay quantifies RBD-specific antibody concentrations using IgG-specific RBD monoclonal antibodies; the full protocols used for eluting DBS samples for the ELISA have been described (*22*). Validation of this test was originally based on PCR-positive infections and prepandemic samples from Boston. To determine an appropriate positivity threshold and assess assay specificity, we measured background antibody reactivity using 104 DBS samples collected in Juba in 2015 (*23*). We then selected a seropositivity threshold (0.32 μg/mL) that corresponded to 100% specificity in these prepandemic samples from Juba (i.e., their highest value; Appendix Figure 1) and 99.7% in the prepandemic samples collected from Boston.

### Statistical Analysis

To estimate test sensitivity, we used data from a cohort of case-patients in Boston with mild and severe confirmed SARS-COV-2 infections whose antibody concentrations had been characterized at multiple time points after symptom onset (*21*) and supplemented these with recent data collected by DBS samples from nonhospitalized PCR-positive patients in Boston (Appendix Figure 2). On the basis of the trends in positive RT-PCR results in Juba, we assumed that most serosurvey participants, if previously infected, would have been exposed to SARS-CoV-2 at least 30 days before the survey (Figure 1) and restricted the positive-control data to observations >30 days after symptom onset during the follow-up period (Appendix Figure 2). Because infections with mild disease may lead to lower levels of detectable antibodies (M.J. Peluso et al., unpub. data, https://doi.org/10.1101/2021.03.03.21251639), we created synthetic cohorts of positive survey participants so that 80% of the sample had mild infections (defined as not needing hospitalization) and 20% had severe cases (defined as hospitalized, but excluding those that died), a proportion consistent with previous analyses (*24*,*25*) and the predominantly young population in Juba (*26*). From 1,000 resampled participants from positive control cohorts, we estimated an average test sensitivity of 65.5%. To evaluate the impact of our assumptions, we also performed sensitivity analyses testing a range of percentages for assumed mild cases (60%–100%) in the positive control dataset.

To estimate the seroprevalence (proportion of the population previously infected), we followed a previously published Bayesian approach (*27*) using a regression model that accounted for age and sex of the study population integrated with a binomial model of the sensitivity and specificity of the ELISA. We selected a random sample from the 1,000 synthetic positive control datasets in each iteration of the model. This approach allowed us to adjust the estimates for test performance while propagating uncertainty around test performance in the adjusted estimates. We did not adjust the estimates for clustering within households because of challenges the field team faced in applying the strict household definition described above. We implemented the models in the Stan probabilistic programming language (https://mc-stan.org) (*28*) using the rstan package in R (https://cran.r-project.org). We poststratified our modeled results, accounting for the age distribution of urban populations in South Sudan (*26*) to generate population-representative seroprevalence estimates. Unless otherwise indicated, we reported estimates as the mean of the posterior samples and 95% credible intervals (CrI) as the 2.5th and 97.5th percentiles of this distribution.

In addition, we used the posterior draws for each regression coefficient to calculate by age and sex the relative risk of participants being seropositive. We used a log-binomial regression model to estimate the relative risk of being seropositive among nonworking adults compared with working adults, children, and students. We estimated implied infections by multiplying our estimated seroprevalence percentage by 510,000, Juba’s estimated 2020 population size (*29*). We then estimated the ratio of reported to unreported infections by subtracting PCR-confirmed SARS-CoV-2 infections from total implied infections in Juba as of August 31, 2020, allowing for a roughly 2-week delay between infection and a seropositive result (*21*), and divided this estimate of unreported infections by the number of RT-PCR–confirmed SARS-CoV-2 infections. The analysis code we used is available online (https://github.com/HopkinsIDD/juba-sars-cov-2-serosurvey), and additional methods are provided (Appendix).

## Results

We recruited a total of 2,214 participants 1–84 years of age from 435 households and provided DBS samples taken during August 10–September 11, 2020. We had complete interview and demographic data for 1,840 (83.2%) but were missing interview data for 374 because of data collection device failures or data entry issues. Of the 1,840 participants, 62.4% were female and 73.5% were 10–49 years of age (Table 1). Both figures were slightly higher than for those same measures from a previous population-representative malaria indicator survey conducted in South Sudan in 2017 (*26*). During April 1–September 11, 2020, a total of 23 deaths (10 male, 13 female) were reported for residents 1–78 years of age within 18 households. None of these deaths were associated with confirmed COVID-19, but 5 patients were reported to have had acute respiratory illness.

**Table 1:** Characteristics of participants with interview data available (n = 1,840) from survey of seroprevalence of SARS-CoV-2 IgG in Juba, South Sudan*

Characteristic	No. (%)	
Sex		
F	1,149 (62.4)	
M	691 (37.6)	
Age, y		
1–4	68 (3.7)	
5–9	224 (12.2)	
10–19	448 (24.3)	
20–29	459 (24.9)	
30–39	307 (16.7)	
40–49	139 (7.6)	
50–64	120 (6.5)	
>65	75 (4.1)	
Payam		
Northern Bari	788 (42.8)	
Juba	141 (7.7)	
Muniki	397 (21.6)	
Kator	229 (12.4)	
Rejaf	135 (7.3)	
Gondokoro	150 (8.2)	
Occupation		
None	408 (22.2)	
Child	386 (21.0)	
Student	388 (21.1)	
Market merchant	89 (4.8)	
Healthcare worker	12 (0.7)	
Taxi driver	16 (0.9)	
Farmer	164 (8.9)	
Working with animals	10 (0.5)	
Civil servant	120 (6.5)	
Health laboratory worker	2 (0.1)	
Teacher	20 (1.1)	
Traditional healer	1 (0.1)	
Religious leader	8 (0.4)	
Other	216 (11.7)	
Reported test for SARS-CoV-2		
No	1816 (98.7)	
Yes	22 (1.2)	
Unknown	2 (0.1)	
Reported SARS-CoV-2 test results		
Negative	15 (0.8)	
Positive	5 (0.3)	
Unknown	2 (0.1)	

*SARS-CoV-2, severe acute respiratory syndrome coronavirus 2.

We found that 22.3% (494/2214) of samples collected during the survey were above the test positivity threshold, which we selected to have 100% specificity against prepandemic samples from Juba. After adjusting for test sensitivity, we estimated that seroprevalence was 38.3% (95% CrI 31.8%–46.5%) in August 2020. This estimate was based on samples from participants with matched interview data available. Seroprevalence in the full dataset was nearly indistinguishable from that in the age- and sex-matched dataset (Appendix Table 3), so we used the latter for all subsequent analyses. These results implied that, for each RT-PCR–confirmed SARS-CoV-2 infection tested by the end of August, 103 (95% CrI 86–126) SARS-CoV-2 infections were unreported.

We found no difference in the risk of seropositivity by sex (Table 2). Seroprevalence was highest at 44.9% (95% CrI 36.3%– 56.0%) among participants 10–19 years of age, a 36% higher risk of being seropositive than among participants 20–29 years of age (RR 1.36, 95% CrI 1.11–1.66) (Table 2). However, uncertainty intervals around seroprevalence estimates by age group were large. In addition, nonworking adults had 35% lower risk (RR 0.65, 95% confidence interval 0.50–0.82) of being seropositive compared to working adults, children, and students. Of the seropositive participants, only 5% reported having had a respiratory illness after April 1, 2020 (Appendix Tables 1, 2). We found no notable relationships between seropositivity and other potential SARS-CoV-2 risk factors (Appendix Table 1).

**Table 2:** Crude seropositivity, adjusted seroprevalence, and relative risk of seropositivity by age and sex from survey of seroprevalence of SARS-CoV-2 IgG in Juba, South Sudan.*

Category	No.	No. (%) positive	No. (%) negative	Seroprevalence (95% CrI)	Relative risk (95% CrI)
Overall	1,840	411 (22.3)	1,429 (77.7)	38.3 (31.8–46.5)	
Age, y					
1–4	68	20 (29.4)	48 (70.6)	43 (31.3–56.1)	1.30 (0.96–1.71)
5–9	224	52 (23.2)	172 (76.8)	39.3 (29.5–51.1)	1.19 (0.92–1.51)
10–19	448	124 (27.7)	324 (72.3)	44.9 (36.3–56)	1.36 (1.11–1.66)
20–29	459	89 (19.4)	370 (80.6)	33.3 (25.6–42)	Referent
30–39	307	52 (16.9)	255 (83.1)	30 (21.9–39.3)	0.91 (0.68–1.17)
40–49	139	26 (18.7)	113 (81.3)	33.2 (22.8–45.6)	1.00 (0.71–1.35)
50–64	120	31 (25.8)	89 (74.2)	42.8 (30.6–57.6)	1.29 (0.94–1.73)
65–84	75	17 (22.7)	58 (77.3)	38.8 (25.2–54.8)	1.17 (0.78–1.63)
Sex					
F	1,149	260 (22.6)	889 (77.4)	33.3 (25.6–42)	Referent
M	691	151 (21.9)	540 (78.1)	31.7 (23.6–41.2)	0.95 (0.81–1.12)

*NA, not available; SARS-CoV-2, severe acute respiratory syndrome coronavirus 2.

**Figure 2:**
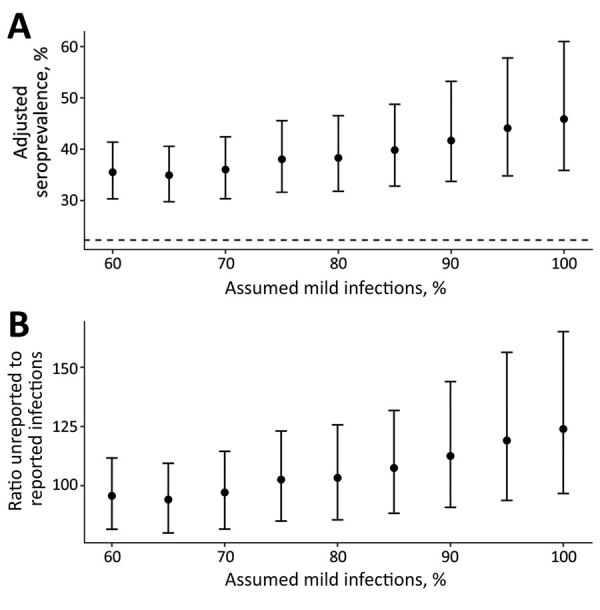
Effects of changing percentage of assumed mild cases in the population on adjusted seroprevalence of severe acute respiratory syndrome coronavirus 2 IgG in Juba, South Sudan. A) Mean adjusted seroprevalence; B) ratio of unreported to reported infections. Error bars represent 95% credible intervals. Dashed line in panel A represents unadjusted seropositivity at 22.3%. Unreported infections in panel B based on 1,873 confirmed coronavirus disease cases in Juba (as of August 31, 2020) and an approximate population of 510,000 in Juba. The *x*-axis in both panels indicates percentage of mild cases included in the synthetic positive control dataset used to estimate assay sensitivity.

We examined potential sources of uncertainty in our estimates. We found higher background levels of antibody reactivity to the SARS-CoV-2 spike protein RBD in prepandemic samples from Juba compared to prepandemic samples from Boston (Appendix Figure 3) (*21*). Since serological measurements from PCR-confirmed cases in Juba were not available, we could not examine whether there were also differences in postinfection antibody dynamics between the populations. However, we were able to assess the impact that different assumptions about test sensitivity had on the results. If we assumed that 60% of infections in the population were mild, we estimated 35.5% (95% CrI 30.3%–41.4%) seroprevalence (Figure 2, panel A) and that, for each reported case, 96 (95% CrI 82–112) cases were unreported (Figure 2, panel B). In contrast, if we assumed that 100% of infections were mild, we estimated 45.9% (95% CrI 35.9%–61.0%) seroprevalence (Figure 2, panel A) and that, for each reported case, 124 (95% CrI 97–165) were unreported (Figure 2, panel B). Regardless of assumptions, these results indicated that 98%–99% of infections through September 2020 had been unreported.

## Discussion

In this study, we estimated that one third of residents of Juba, South Sudan had been infected with SARS-CoV-2 through September 2020. That proportion corresponds to ≈196,000 implied infections, >100 times the number of PCR-confirmed SARS-COV-2 infections over the same time frame. These results reveal that in Juba, similar to in other sub-Saharan Africa populations, although the COVID-19 pandemic has had less apparent health impact than in other parts of the world, the virus has spread extensively.

Adjusting for imperfect immunoassay performance is critical when estimating infection attack rates from serosurveys. Postinfection antibody kinetics vary by infection severity, patient age, and prior exposure, as can test performance. When we tested prepandemic samples from Juba, we found that background SARS-CoV-2 antibody reactivity was higher in Juba than in Boston, which was consistent with findings from studies conducted in other sites in sub-Saharan Africa (*11*,*13*,*30*,*31*). We used these negative controls to estimate test specificity, but we lacked data on the post SARS-CoV-2 infection antibody kinetics and the proportion of infections that were mild or asymptomatic in the Juba population, which led to wide variation in plausible estimates of seroprevalence, as shown in our sensitivity analyses.

Our findings have several implications for SARS-CoV-2 control in South Sudan. At least one third of the population in Juba has been exposed to the virus, and this proportion undoubtedly has increased since the survey was completed in September 2020. The low proportion of seropositive patients reporting respiratory symptoms suggests that the overwhelming majority of these infections were mild or asymptomatic. These estimates will help public health decision makers in South Sudan weigh the costs and benefits of devoting limited resources to COVID-19 mitigation at the cost of other crucial health programs.

One question we were unable to address was whether transmission occurred predominantly within households. However, crowded living conditions among Juba’s urban population, including 31.3% of households living in 1- or 2-room shelters and 19.5% of households having >4 members sleeping in the same room, support this hypothesis (*20*). Another unanswered question is the extent to which SARS-CoV-2 spread and mitigation measures have exacerbated underlying vulnerabilities, including food insecurity, livelihoods, and co-infections, such as the current measles outbreak in South Sudan (*32*). Follow-up studies would be required to understand the larger impact of the epidemic in Juba as well as in the rest of South Sudan and to better inform public health policy.

These results also have implications for SARS-CoV-2 serosurveillance more broadly. Most serosurveys conducted to date, if they adjust seroprevalence estimates for test performance at all, use sensitivity and specificity estimates provided by assay manufacturers, which may be overly optimistic and based on a narrow range of samples (*6*). In many settings it may not be feasible to collect control data from local populations, but validating different immunoassays in populations in the same region of the world where the assays are being used is critical for appropriate interpretation of study results. Moreover, our findings support previous studies that have called for including mild and asymptomatic SARS-CoV-2 infections in assay validation datasets (*33*). We and others have shown that antibody titers from mild and asymptomatic infections tend to be lower (*34*–*39*). Thus, validation datasets comprised predominantly of data from severe, hospitalized cases may lead to overestimating assay sensitivity and gross underestimation of SARS-CoV-2 seroprevalence (*33*). 

Overall, the SARS-CoV-2 seroprevalence estimates reported in this study are comparable to estimates in Nigeria of 25%–45%, depending on the population sampled (*8*,*10*; H. Majiya et al., unpub. data, https://doi.org/10.1101/2020.08.04.20168112). Similarly, seroprevalence was 40% in public sector patients in Cape Town, South Africa (*14*), 12.3% among asymptomatic healthcare workers in Blantyre, Malawi (*12*), and 25.1% among gold mine workers in Côte d’Ivoire (*15*). In Addis Ababa, Ethiopia, seroprevalence among those reporting no close contact with SARS-CoV-2 infected persons was 8.8% in April 2020 (B.N. Alemu et al., unpub. data, https://doi.org/10.1101/2020.10.13.337287). Seroprevalence was lower at 4.3% in blood donors in Kenya in June 2020 (*7*), increasing to 9.1% by September (I.M.O. Adetifa et al., unpub. data, https://doi.org/10.1101/2021.02.09.21251404), and was 10.6% in 6 districts in Zambia in July 2020 (*16*). These lower estimates may be due to differences in SARS-CoV-2 epidemiology, time periods included, or subpopulations measured. Serologic tests may themselves contribute to differences. A study in Kinshasa, Democratic Republic of the Congo, showed that seropositivity in health facility staff was 8%–36% depending on the serological test used (*13*). Nevertheless, findings from these studies taken together indicate that SARS-CoV-2 has spread widely in sub-Saharan Africa (*2*,*3*). This conclusion is supported by a postmortem study in Lusaka, Zambia, which found that among 372 deceased patients, 19.2% were PCR-positive for SARS-CoV-2 (*40*).

One of the limitations of our study is that, as we have described, our positive control data came from a cohort in Boston. Therefore, despite our efforts to correct for differences between the populations, we do not know how accurate our sensitivity estimates are for Juba or elsewhere in Africa. In addition, we used a single ELISA that measured IgG targeting the RBD of SARS-CoV-2’s spike protein. Previous studies have shown variation in sensitivity and specificity of antibody assays that target different antigens (*13*,*41*), suggesting that testing for multiple antigens may provide a better picture of seroprevalence than those targeting a single antigen alone, particularly when validation data are not available from the local population. Although the study had a standard definition for households, the study team faced challenges in following this strict definition; as a result, we were unable to confidently estimate the degree to which SARS-CoV-2 infections clustered within households, nor could we adjust for these variations in the regression model. This difficulty also prevented us from calculating mortality rates based on reported household deaths. Finally, whereas this study was representative of the residential neighborhoods of Juba, the sample did not include an estimated >30,000 IDPs living in 2 camps in Juba (*42*). Nevertheless, 14.3% of households participating in the study self-reported as IDPs, either living in the host community or at another IDP site.

Our study’s strengths include that it is one of few population-based seroprevalence studies conducted in and representative of the general population of a city or other geographic region within sub-Saharan Africa. Furthermore, we used specificity estimates based on background antibody levels specific to the local population, adjusted seroprevalence estimates based on test results, and propagated uncertainty around test performance into our final estimates. Because the ELISA we used was quantitative, we reported antibody distributions rather than seropositivity cutoffs alone (Appendix Figure 1). As a result, it would be possible to adjust our estimates further if more accurate sensitivity data become available for this population.

In conclusion, we present evidence that SARS-CoV-2 seroprevalence is much higher in Juba than suggested by confirmed case data alone, which is consistent with findings from other recent serosurveys in sub-Saharan Africa. Future serosurveys in South Sudan will be helpful to confirm these findings and to examine the effect that SARS-CoV-2 spread has had on underlying vulnerabilities. Such seroprevalence studies are needed to understand the impact of the pandemic more broadly in Africa, as well as the ways to most effectively mitigate its effects. For these efforts to be most effective, however, they must be accompanied by efforts to validate serologic tests in local populations.

Appendix Additional information on survey of seroprevalence of SARS-CoV-2 IgG in Juba, South Sudan.
