# Supplementary material for: Seroprevalence of Severe Acute Respiratory Syndrome Coronavirus 2 IgG in Juba, South Sudan, 2020
Source: Emerg Infect Dis. 2021 Jun;27(6):1598–606. doi: 10.3201/eid2706.210568 (PMC8153877; doi:10.3201/eid2706.210568)
Supplement: Appendix — Additional information on survey of seroprevalence of SARS-CoV-2 IgG in Juba, South Sudan. [file 21-0568-Techapp-s1.pdf]

Article DOI: https://doi.org/10.3201/eid2706.210568

Seroprevalence of Severe Acute Respiratory Syndrome Coronavirus 2 IgG in Juba, South Sudan, 2018–2019

Appendix

Statistical Model

We aimed to estimate the true underlying seroprevalence of SARS-CoV-2 in the population ≥1 year of age in Juba, South Sudan. To that end, we estimated the probability that each participant in the serosurvey was seropositive using a Bayesian logistic regression model (*1*) that accounts for serologic test sensitivity and specificity, as well as age and sex of each participant:

$$x^i \approx Bernoulli(p_i\theta^+ + (1 - p_i) * (1 - \theta^-))$$

$$logit(p_i) = X_i\beta$$

$$x^+ \approx Binomial(n^+, \theta^+)$$

$$x^- \approx Binomial(n^-, 1 - \theta^-)$$

Here x^i was the result of the IgG ELISA for each individual (i = 1,…, N = 1,840) in the serosurvey. The probability of observing a seropositive result was a function of sensitivity, θ^+ (true positive rate), and specificity, θ^- (true negative rate), in the context of the true underlying probability of seropositivity for each individual, p_i. This probability p_i was a function of covariates X_i, which included the age and sex of each individual, and their coefficients β. Sensitivity, θ^+, was determined using n^+ RT-PCR–confirmed positive controls from the Boston cohort (*2*) (Appendix Figure 2; methods in the main text). Specificity, θ^-, was determined using prepandemic negative controls (*3*), where x^- tested positive. Priors on sensitivity and specificity were flat from 0 to 1 and priors on regression coefficients β were $Normal(0,1)$.

We implemented the model in the Stan probabilistic modeling language (https://mc-stan.org) (*4*) using the *rstan* package in R (https://cran.r-project.org). We ran 5,000 total iterations, which included 4 chains with 1,500 iterations each and 250 for warm-up. The complete modeling and analysis code is available online (https://github.com/HopkinsIDD/juba-sars-cov-2-serosurvey).

References

1. Stringhini S, Wisniak A, Piumatti G, Azman AS, Lauer SA, Baysson H, et al. Seroprevalence of anti-SARS-CoV-2 IgG antibodies in Geneva, Switzerland (SEROCoV-POP): a population-based study. Lancet. 2020;396:313–9. PubMed https://doi.org/10.1016/S0140-6736(20)31304-0

2. Iyer AS, Jones FK, Nodoushani A, Kelly M, Becker M, Slater D, et al. Persistence and decay of human antibody responses to the receptor binding domain of SARS-CoV-2 spike protein in COVID-19 patients. Sci Immunol. 2020;5:eabe0367. PubMed https://doi.org/10.1126/sciimmunol.abe0367

3. Iyer AS, Azman AS, Bouhenia M, Deng LO, Anderson CP, Graves M, et al. Dried blood spots for measuring *Vibrio cholerae*-specific immune responses. PLoS Negl Trop Dis. 2018;12:e0006196. PubMed https://doi.org/10.1371/journal.pntd.0006196

4. Carpenter B, Gelman A, Hoffman MD, Lee D, Goodrich B, Betancourt M, et al. Stan: a probabilistic programming language. J Stat Softw. 2017;76:1–32. https://doi.org/10.18637/jss.v076.i01

Appendix Table 1. History of respiratory illness and SARS-CoV-2 serostatus in participants with interview data available (n = 1,840) from study of seroprevalence of SARS-CoV-2 IgG in Juba, South Sudan.

Characteristic	N (%)*	Seropositive (%)†
Payam		
Northern Bari	788 (42.8)	157 (19.9)
Muniki	397 (21.6)	97 (24.4)
Juba	141 (7.7)	34 (24.1)
Kator	229 (12.4)	77 (33.6)
Rejaf	135 (7.3)	23 (17.0)
Gondokoro	150 (8.2)	23 (15.3)
Occupation		
None	408 (22.2)	64 (15.7)
Student	388 (21.1)	89 (22.9)
Child	386 (21.0)	105 (27.2)
Teacher	20 (1.1)	3 (15.0)
Farmer	164 (8.9)	34 (20.7)
Market merchant	89 (4.8)	17 (19.1)
Civil servant	120 (6.5)	29 (24.2)
Religious leader	8 (0.4)	4 (50.0)
Health laboratory worker	2 (0.1)	1 (50.0)
Taxi driver	16 (0.9)	1 (6.2)
Healthcare worker	12 (0.7)	1 (8.3)
Working with animals	10 (0.5)	4 (40.0)
Traditional healer	1 (0.1)	0 (0.0)
Other	216 (11.7)	59 (27.3)
Reported test for SARS-CoV-2		
No	1,816 (98.7)	407 (22.4)
Yes	22 (1.2)	3 (13.6)
Unknown	2 (0.1)	1 (50.0)
Reported SARS-CoV-2 test result		
Negative	15 (0.8)	2 (13.3)
Positive	5 (0.3)	0 (0.0)
Unknown	2 (0.1)	1 (50.0)
Respiratory illness		
No	1,727 (93.9)	389 (22.5)
Yes	113 (6.1)	22 (19.5)
Respiratory illness month		
April	10 (0.5)	3 (30.0)
May	15 (0.8)	4 (26.7)
June	34 (1.8)	8 (23.5)
July	29 (1.6)	4 (13.8)
August	25 (1.4)	3 (12.0)
Sought medical care for illness		
No	34 (1.8)	9 (26.5)
Yes	79 (4.3)	13 (16.5)
Missed work or school for illness		
No	66 (3.6)	11 (16.7)
Yes	38 (2.1)	8 (21.1)
Unknown	9 (0.5)	3 (33.3)
Hospitalized for illness		
No	86 (4.7)	13 (15.1)
Yes	25 (1.4)	8 (32.0)
Unknown	2 (0.1)	1 (50.0)
Traveled in South Sudan		
No	1,818 (98.8)	407 (22.4)
Yes	20 (1.1)	3 (15.0)
Unknown	2 (0.1)	1 (50.0)
Traveled internationally		
No	1,834 (99.7)	410 (22.4)
Yes	6 (0.3)	1 (16.7)
COVID-19 contact		
No	1,765 (95.9)	395 (22.4)
Yes	4 (0.2)	1 (25.0)
Unknown	71 (3.9)	15 (21.1)
Acute respiratory illness contact		
No	1,703 (92.6)	382 (22.4)
Yes	54 (2.9)	12 (22.2)
Unknown	83 (4.5)	17 (20.5)

Characteristic	N (%)*	Seropositive (%)†
Attended a large gathering		
No	1,529 (83.1)	342 (22.4)
Yes	304 (16.5)	68 (22.4)
Unknown	7 (0.4)	1 (14.3)
Visited a hospital		
No	1,765 (95.9)	394 (22.3)
Yes	71 (3.9)	17 (23.9)
Unknown	4 (0.2)	0 (0.0)
Visited a traditional healer		
No	1,834 (99.7)	409 (22.3)
Yes	3 (0.2)	1 (33.3)
Unknown	3 (0.2)	1 (33.3)

*N is total number of participants included in each category and % indicates percentage of the participants that fell within each category.
†Seropositive is the number of participants with antibody titers above the seropositivity threshold, and % is the percent of participants within each group that were seropositive.

Appendix Table 2. Symptoms and SARS-CoV-2 serostatus among participants reporting a respiratory illness (n = 113) during April 1–September 11, 2020, from study of seroprevalence of SARS-CoV-2 IgG in Juba, South Sudan.

Symptom	N (%)*	Seropositive (%)†
Cough	73 (64.6)	16 (21.9)
Runny nose	64 (56.6)	17 (26.6)
Fever	29 (25.7)	7 (24.1)
Joint pains	29 (25.7)	6 (20.7)
Sore throat	21 (18.6)	5 (23.8)
Headache	18 (15.9)	2 (11.1)
Chest pain	15 (13.3)	3 (20.0)
Wheezing	13 (11.5)	3 (23.1)
Loss of appetite	10 (8.8)	2 (20.0)
Fatigue	8 (7.1)	1 (12.5)
Muscle aches	7 (6.2)	2 (28.6)
Shortness of breath	6 (5.3)	1 (16.7)
Vomiting	6 (5.3)	0 (0.0)
Loss of or altered taste	5 (4.4)	2 (40.0)
Loss of or altered smell	4 (3.5)	2 (50.0)
Chills	3 (2.7)	1 (33.3)
Nausea	3 (2.7)	0 (0.0)
Abdominal pain	1 (0.9)	1 (100.0)
Diarrhea	1 (0.9)	1 (100.0)
Red eyes	1 (0.9)	0 (0.0)
Nose bleeding	1 (0.9)	0 (0.0)
Other	5 (4.4)	0 (0.0)

*N is total number of participants included in each category and % indicates percentage of the participants that fell within each category.
†Seropositive is the number of participants with antibody titers above the seropositivity threshold, and % is the percent of participants within each group that were seropositive.

Appendix Table 3. Adjusted estimates from study of seroprevalence of SARS-CoV-2 IgG in Juba, South Sudan.

Analysis	Seroprevalence, % (95% CrI)
Primary*	38.3 (31.8–46.5)
No covariates‡¶	36.4 (31.2–42.7)
No covariates‡†	36.3 (31.1–42.6)

*Primary analysis includes estimates adjusted for test performance and age and sex of the participants.
†Used the subset that can be matched to age and sex data (n = 1840).
‡"No covariates" analysis includes estimates adjusted for test performance alone.
¶Used full serologic dataset (n = 2214).

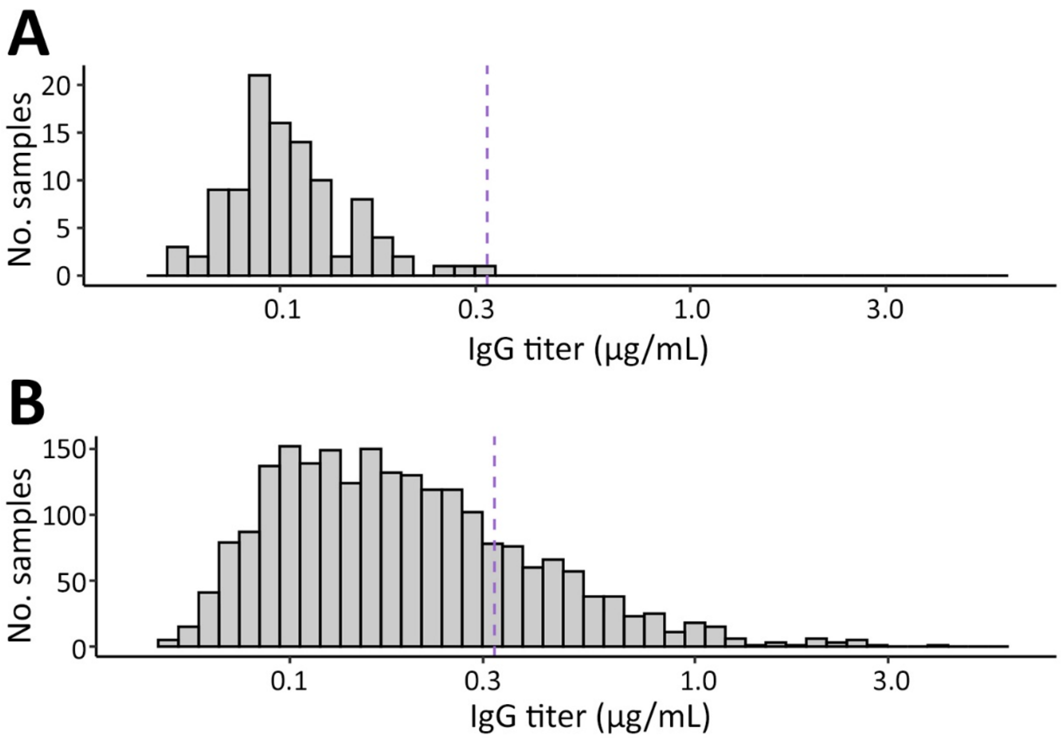

Appendix Figure 1. Distribution of anti-SARS-CoV-2 antibodies in the Juba population A) in 2015 before the pandemic (n = 104) and B) during the survey (n = 2,214) from study of seroprevalence of SARS-CoV-2 IgG in Juba, South Sudan. Histograms of IgG titers A) in 2015 before the pandemic and B) in 2020 during the survey. The dashed line indicates the maximum value detected in any prepandemic sample (0.32 µg/mL), which we used as the seropositivity cutoff.

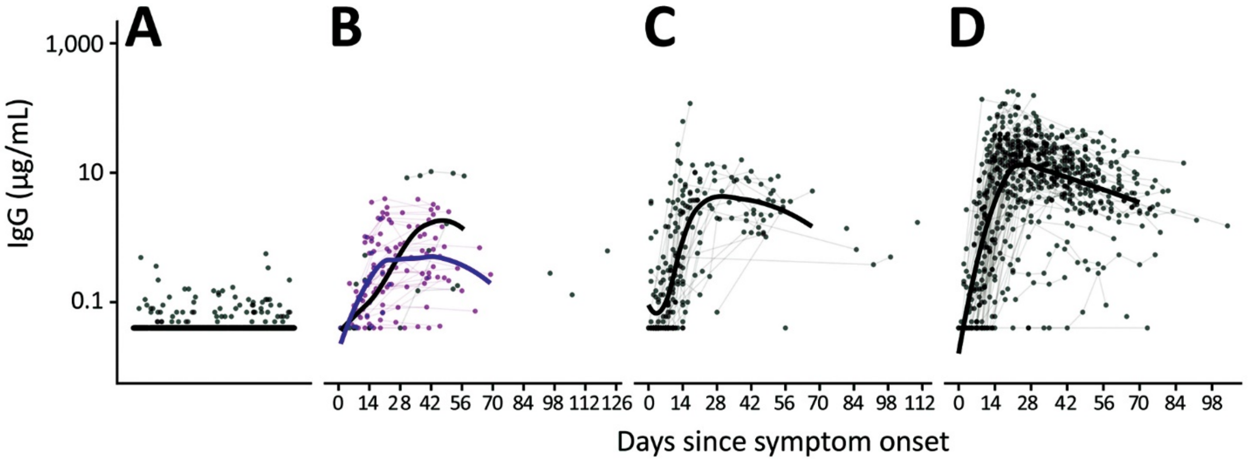

Appendix Figure 2. Antibody dynamics in Boston, Massachusetts, United States cohort from study of seroprevalence of SARS-CoV-2 IgG in Juba, South Sudan. Panels show A) prepandemic samples (controls); B) COVID-19 cases that did not require hospitalization; C) cases that required hospitalization but not intensive care, and D) cases that were hospitalized and required intensive care. Black points represent individual IgG titers at various time points after symptom onset; time points for individual COVID-19 cases are connected by gray lines. The limit of detection of the serologic test was 0.04 µg/mL. Black points and smoothed trajectories for days 0–70 represent data from Iyer et al. 2020 (*2*). Purple points and trajectories represent additional data from mild PCR-confirmed COVID-19. Data from patients who died are not included.

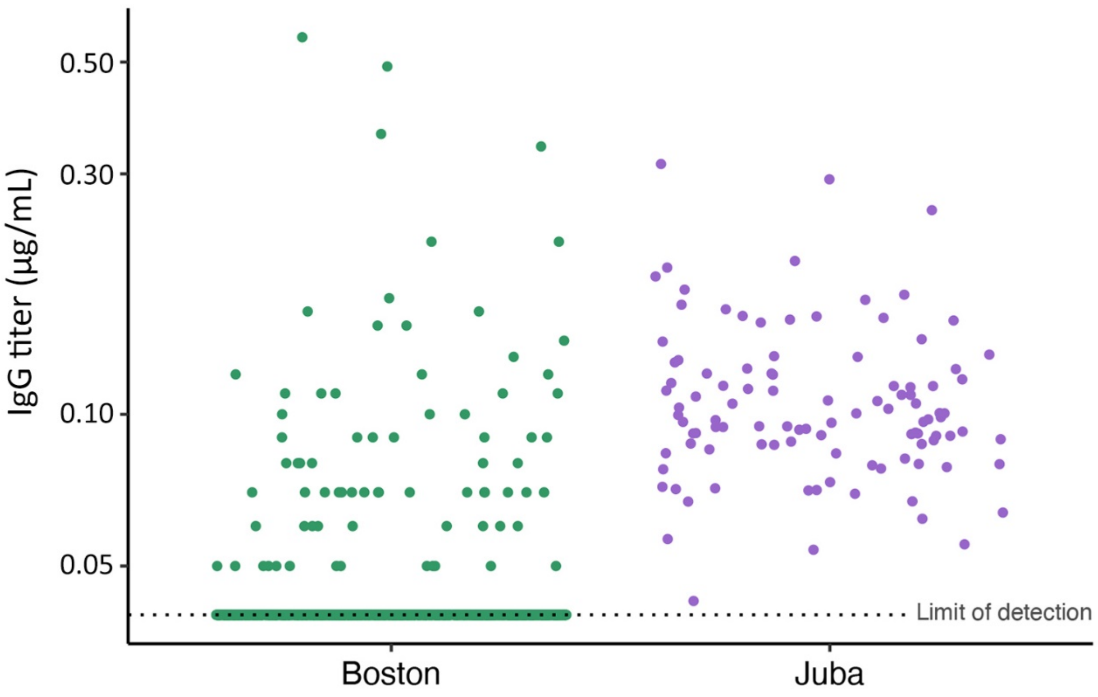

Appendix Figure 3. Antibody distributions in prepandemic negative controls from populations in Boston, Massachusetts, United States (n = 1,548) and Juba, South Sudan (n = 104) from study of seroprevalence of SARS-CoV-2 IgG in Juba, South Sudan. Each point represents an individual test result. Dotted line represents the limit of detection of the serologic test. Boston data collected before the pandemic are shown in green and represent a combination of healthy adults seen at the Massachusetts General Hospital travel clinic, patients undergoing routine serology testing at Massachusetts General Hospital, and patients presenting with a known febrile illness. The green line at the limit of detection indicates that most of samples from Boston had background reactivity that fell below this limit. Data collected from Juba in 2015 are shown in purple; none of the Juba samples fell below the limit of detection.
